# Re‐establishing and preserving hope of recovery through user participation in patients with a severe mental disorder: the self‐referral‐to‐inpatient‐treatment project

**DOI:** 10.1002/nop2.59

**Published:** 2016-06-28

**Authors:** Silje S. Samuelsen, Inger Elise Opheim Moljord, Lasse Eriksen

**Affiliations:** ^1^ St Olavs University HospitalNidaros Community Mental Health CenterTrondheimNorway; ^2^ Norwegian University of Science and TechnologyFaculty of MedicineInstitute of NeuroscienceTrondheimNorway

**Keywords:** hope, person‐based approaches, recovery, regulation of emotions, self‐referral bed, severe mental disorder

## Abstract

**Aims:**

The treatment of patients with a severe mental disorder is generally not good enough. The aim of this article was to illustrate some alternative approaches for better understanding and treatment for the individual, besides seeing and interpreting the symptoms.

**Methods:**

The context of understanding is regulation of emotions whit a person‐based approach. The self‐referral‐to‐inpatient‐treatment project is presented and discussed as a possible method of intervention to improve patient involvement.

**Design:**

Theoretical approach.

**Results:**

Involvement in genuine decisions, where the individual is in control and feels emotionally robust, is important. The experience of regaining authority through being self‐empowered with sufficient environmental support is essential for re‐establishing and preserving hope of recovery.

## 1 Introduction

Mental health services should as far as possible provide voluntary and independent self‐asserting treatment based on the ability to cope with one's own life. Patients are commonly not sufficiently included in their own treatment, and increased patient involvement continues to be an important goal (Health and Social Affairs 2004; Lester, Tait, England, & Tritter, 2006; Longtin et al., 2010). Patient involvement can motivate and facilitate patients to use their rights to take an active part in their recovery (Rise et al., 2011; Storm & Edwards, 2013; WHO 2009). These approaches should be structured in a way that enables the patients to make use of their own resources and actively contribute to their own life (Gudde et al., 2013). Unfortunately, mental treatment is generally not good enough for people with long‐term and complex mental disorders (Health and Social Affairs 2006). The Department of Health and Care's Commission Document for the Central Norway Regional Health Authority (2010) emphasized that the self‐referral‐to‐inpatient‐treatment (SRIT) program could be a method used to increase patient participation and reduce involuntary admissions. The rehabilitation unit at a community mental health (CMHC) center in Norway established in 2010 a SRIT project for measuring the effect of it. To discuss the purpose of the project, we will present a theoretical framework based on the understanding of severe mental disorders, difficulties with regulation and theory of recovery and in addition give a presentation of the research project at CMHC. The aim of this study was to present how a new approach to severe mental disorders can contribute to re‐establishing and preserving hope of recovery through user participation.

## 2 Background

### 2.1 Severe mental disorder

The criterion for participation in the SRIT project was a severe mental disorder. This was defined as a psychotic or bipolar disorder with or without drug problems. A total of 74% participants with a schizophrenia diagnosis were included. Self‐injury or mutilations, acting out, massive drug abuse or lack of ability to see the need for and function of the SRIT project were criteria for exclusion. Traditionally, it was supposed that participants probably would be in need of long‐term service from primary and secondary care.

The psychological condition varies from person to person, because psychosis and bipolar disorder are viewed as generalized diagnoses for a collection of conditions with similar symptoms. The condition afflicts the most basic mental functions that contribute to such aspects as a feeling of self, individuality and purpose (Ralph & Corrigan, 2005). A common trait might be that these conditions lead to a negative development in the patients' psycho‐social functioning (Hagen, Berge, & Gråwe, 2007).

Empirical studies have clearly shown that experiences like strange assumptions and hearing voices are aspects of a continuum in which it is difficult to distinguish normality from abnormality. Experiences like these are not isolated to symptoms of psychosis but rather common human experiences (Hagen et al., 2007). As a therapist, one of the greatest challenges is how to approach people with severe mental disorder. The diagnosis can only describe something in general about the condition. By viewing the behavior as unique for each individual, one can get an understanding of how the symptoms function and get a clearer picture of the suffering of each person (Chadwick, 2006).

### 2.2 Regulation of emotions as a framework of understanding

The patient's emotional control can be viewed as a gateway to understand symptoms. Emotional control is defined as processes that reflect how well one masters emotional triggering and adapts to social and non‐social responses. Regulation is assumed at every level of the emotional processes, each time an emotion is triggered, and even before an emotion manifests itself (Campos, Frankel, & Camras, 2004).

Emotional control can be understood as a tool by which the emotions organize attention and actively facilitate strategies such as taking actions to solve a problem. This enables the person to maintain a sense of wellbeing, while at the same time creating room for reflection, planning and acting in accordance with the surrounding world (Cole, Martin, & Dennis, 2004). Overstimulation and disrupted emotions are seen as the basis for bodily uneasiness and can lead to relapses of disease (Gumley & Schwannauer, 2006). Similar to those with mood disorders and serious anxiety, people with psychosis are bothered by their emotions. They use a lot of energy trying to understand and regulate themselves (Gudde et al., 2013; Hatfield & Lefley, 1993), and their emotional control is often a maladaptive art. Difficulties in over‐ and under‐regulation of emotions often lead to significant problems (Greenberg, 2002).

Emotions are an important source to understand a person. By observing how persons react to various life events, knowledge will be provided. Emotional control can tell us how emotional organization facilitates other psychological processes such as attention, problem solving and relations to other people (Cole et al., 2004). Environmental vulnerability in people with severe mental disorder can result in maladaptive responses to stressful situations. Learning to adaptively regulate oneself, instead of trying to avoid emotions when uneasiness occurs, is therefore seen as an important gateway to recovery (Campos et al., 2004). An important aspect in the development of social abilities is to express and tolerate emotions. Establishing a secure environment with close relations where one is empathically engaged can be seen as a buffer in the regulation.

### 2.3 User participation as part of the recovery process:

Recovery can be viewed as regaining previous functions as well as being more psychologically robust than before. Recovery is defined in the study as reflection of the subjective attitudes and orientations in which the individuals, regardless of disorder, can hope to be able to expand their personal abilities and make their own choices.

It is essential for the recovery process that the patient regains a feeling of self by redefining the disorder from being ‘something one is’ to ‘something one has’ (Hagen et al., 2007; Torgalsbøen, 2005). This contributes to the possibility that the subjective attitude towards oneself will be independent of the stage of the disorder. How the persons view themselves, relate to their hopes and expectations of life, and compare themselves to other people is essential. Studies have shown that a long progression of disorder often leads one to an acceptance of one's own chronic state (Priebe & Fakhoury, 2008). The level of achievement decreases to an obtainable level (Chadwick, 2006; Corrigan, Mueser, Bond, Drake, & Solomon, 2008). This is confirmed by studies which show that patients with psychosis are more satisfied with their life than therapists objectively would expect (Priebe & Fakhoury, 2008).

Encouragement and support of the patient interests, talents, vitality and efforts have been shown to contribute to improvement (Borg & Davidson, 2008; Roe & Davidson, 2008; Warner, 2010; Wilken, 2007). Creating a safe environment, listening and trying to understand the person, and maintaining a dialogue and support system can give access to meaningful choices and create a space for taking risks (Gudde et al., 2013; Roe & Davidson, 2008). The significance of being seen, heard, understood, accepted and respected is important for recovery (Borg & Davidson, 2008). These factors will always be an interaction between the internal and the external, something Wilken (2007) refers to as self‐empowering and environmental empowering. As helpers, we may contribute to environmental empowering by offering knowledge, experience and resources for hope and faith (Roe & Davidson, 2008).

A good and stable relationship over time is one of the most important determinants for success in therapy. In user participation, mutual respect, dialogue and decision‐making are fundamental elements for an individual that needs treatment (Rise et al., 2011) and hope for recovery. To maintain important democratic principles, user participation has been a central aspect in Norway since the 1980s. This approach has unfortunately centered on consumerism of voice more than choice (Andreassen, 2009). The rhetoric has largely failed to be translated into practice (Newman, O'Reilly, Lee, & Kennedy, 2015). For participation to be effective, it has been shown that the individual must be involved in genuine decision‐making (Tempfer & Nowak, 2011). The experience of being in control of the situation – self‐empowerment – is seen as essential to the recovery process where hope and regaining of authority is essential (Connell, Brazier, O'Cathain, Lloyd‐Jones, & Paisley, 2012; Torgalsbøen, 2005; Wilken, 2007). The definition and organization of user participation in each treatment is therefore considered important (Rise et al., 2011). The service that is provided should correspond with the user's needs and wishes (Hickey & Kipping, 1998). Our job as helpers is not to serve fish but just to give advice on the art of fishing it. The involvement in a patient's life must be adapted and created to fit the patient's experience of his or her needs. Patient involvement is one of the main measures which is important in an efficient health care service (Tritter, Koivusalo, Ollila, & Dorfman, 2010).

### 2.4 The self‐referral‐to‐inpatient‐treatment project

Effect, process, and experience are included in a randomized controlled trial with quantitative and qualitative methods, approved by the Regional Ethical Committee. The participants were included between 2010–2012. The aim of the SRIT project was to help patients in a worsening period and the rehabilitation unit established to beds for this. The 53 voluntary patients were randomized into two groups: (1) The intervention group, the participants got a contract for SRIT at once. The participants or their relatives could, ask the rehabilitation section at the CMHC directly for a bed without contacting their GP, the emergency department, or the on‐duty doctor. The participants had to contact the section before arrival when they wanted to use SRIT. They could come to the section each day between 08.00 a.m.–20.00 p.m. and before 15.30 p.m. on Friday if they wanted to stay during the weekend. There had to be 14 days between each SRIT stay. If there was need for hospitalization longer than 5 days, the SRIT could be transferred to regular stay. If the participants with a contract showed signs of the exclusion criteria (e.g. intoxicated) and wanted to use the contract of SRIT, they had to use the common admission procedures. If both beds were occupied they had to wait until one was available, between 1‐5 days. After entrance to the unit, all patients had a conversation with the staff. Each stay was characterized by care, rest, structure, nutrition, social support and practical support. The patients' commitment was to follow the department's procedures and activities. One of the objectives of SRIT was to teach the participants ‘to listen to themselves’, their early signs, and take more responsibility of their own lives. The contract in itself could function as a reminder of what to do when the patient's problem increased. They could contact their out‐patient therapist or counselor, a friend or relative, or the department for advice or for ‘booking’ a place. The learning goal for this group was to seek help in time. Contact with known staff members who were open to dialogue and reflection with the patients, in a familiar unit, provide predictability and safety to persons with a major mental disorder. This was found to be important for this group of patients (Gudde et al., 2013). (2) The participants in the treatment as usual (TAU) group had to wait 1 year before they got a contract of SRIT. They had to use common admission procedures if they were in need of hospitalization during that year.

Both quantitative and qualitative data were collected before randomization and after 4, 8, 12 and 24 months, with use of different self‐reported scales and semi‐structured interviews. Register data including involuntary admissions from all the patients was also included. The project group was composed of researchers, user‐researchers, psychologist and psychiatric nurses. User‐researchers were involved when planning the project, making the interview guide, finding meaningful units in the interviews and deciding which issues require extended knowledge through new interviews.

## 3 Discussion

Patient involvement in treatment is described as important for the recovery process (Rise et al., 2011; Tritter et al., 2010; Wilken, 2007). Despite this knowledge, the users are only offered a voice, not a real choice (Andreassen, 2009). The rhetoric has largely failed to be translated into practice (Newman et al., 2015). As a reaction to this, in addition to new governmental guidelines and the fact that randomized clinical trials were missing, the SRIT project was given to CMHC. One qualitative study from the project has compared the experiences of self‐referral inpatient treatment with treatment as usual (Rise et al., 2013). The result from 4 months after randomization indicates that patients with a contract for SRIT had come further in the recovery process. Patient's with a contract expressed less powerlessness, resignation and hopelessness. The users were involved in decisions about managing their own illness. They felt more self‐empowered, developed a positive identity and found meaning in their lives (Olso et al., 2016).

Treatment can be complex if a patient has a long history of disorder. It is important to see and to find ‘the persons behind the disorder’. The persons can, in different ways, protect themselves against emotional discomfort. Environmental empowerment is here seen as a possible facilitator for adaptive regulation. Therefore, the project at CMHC focuses on creating a safe environment with familiar relations that is in dialogue with users, both on a group and individual level. We believe that contact with familiar personnel in a familiar department provides predictability (Gudde et al., 2013). This can reduce stress and help the patients to stabilize their mental state faster than if they were admitted to a department with unfamiliar personnel because the treatment is person based. They are able to decide when they need an inpatient stay and what to focus on during that stay. This resulted in self‐empowerment and a feeling of hope. The interaction between patients and helpers can be challenging, especially with respect to power balance. Health personnel have an ethical responsibility to assure the patients' integrity during treatment in the best way possible. In cases where the lack of function is severe and self‐knowledge is limited, health personnel have a legal responsibility to give adequate and necessary treatment, despite a patient's wishes. This is to ensure that the patient's condition improves and stabilizes so that he or she is not a danger to himself/herself or others. To receive help against one's will be perceived as degrading and can contribute to a negative therapeutic experience. Therapy should be focused on how the helpers can contribute to assisting the patient become self‐empowered. To feel safe enough to challenge oneself and to take chances creates development and gives meaning to life. At the same time, it is important to know that there is a ‘safe port’ to return to.

It can sometimes be difficult for the user to understand the need for admission. The patient and the helper can experience the situation differently. It is therefore vital to communicate respectfully. Decisions should be made, if possible, in accordance with both parties (Rise et al., 2011). One should therefore at an early stage review possible changes and investigates if the user and the helper have similar experiences of improvement. The development of one's own abilities and making one's own choices is essential for sustaining hope and believing in change. Being viewed as more than an disorder and to redefine oneself apart from the disorder is seen as important for the re‐conquering of the self. Building on each patient's interests, talents, energy and efforts contributes to recovery (Roe & Davidson, 2008; Warner, 2010; Wilken, 2007). Humans have a need to be seen for who they are and to feel chosen and respected. Their value has to be seen separate from their achievements (Roe & Davidson, 2008) and services needs to be based on the needs of the individual.

It is important to be aware of the needs of the individual and create a space for taking chances and making mistakes (Gudde et al., 2013; Roe & Davidson, 2008). With adequate support, one can challenge oneself outside the boundaries of one's own comfort zone. Taking risks jeopardizes the mental state, but lack of challenge can also lead to stagnation and negative development. The level of ambition determines the amount of risk one is willing to take. A delicate balance of ambitions exists in the interaction between the user and the helper. A helper can have great ambitions for the user which do not correspond with the user's own wishes and goals. It is therefore essential in an early state to operationalize and define the user's wishes and goals in such a way that the support the helper gives, meets the user's perception of need. In this case, a helper can be a part of challenging the patients by not giving up prior goals or dreams but rather adapting them to the situation the user is in. And through this re‐establishing and preserving hope.

## 4 Conclusion

To re‐establish and preserve hope of recovery, the patient's involvement in treatment is essential. Regulation of emotions as a framework of understanding seems to be a better way than focusing on symptoms. The patient's experience of being in control and feeling emotionally robust contributes to the regaining of authority and being self‐empowered. User participation, as in the self‐referral‐to‐inpatient‐treatment project, can contribute to effective health services that are person based, and therefore takes into account, the person's voice and choice. Our job as helpers is to give adequate environmental empowering and contribute to create hope, such that patients can master their own lives, being self‐empowered and adapting the goals to the situation the user is in, in addition to preserving and re‐establishing hope.

## Funding

St. Olav's University Hospital, Division of Psychiatry, Department of Nidaros CMHC funded the study.

## Ethical approval

Self‐referral‐to‐inpatient beds, was approved by the Regional Committee for Medical Research Ethics (REK) East‐Norway and Norwegian Social Science Data Service (NSD).

## Author contribution

All authors gave important contributions to conceptions and design of this study. Inger Elise Opheim Moljord wrote the beginning and the description of the project. Silje Samuelsen wrote the main theoretical part and discussion. All authors revised it critically for essential intellectual content and gave their final approval of the version to be published.

All authors have agreed on the final version and meet at least one of the following criteria [recommended by the ICMJE (http://www.icmje.org/recommendations/)]:

- substantial contributions to conception and design, acquisition of data, or analysis and interpretation of data;
- drafting the article or revising it critically for important intellectual content.
